# Primary care asthma surveillance: a review of knowledge translation tools and strategies for quality improvement

**DOI:** 10.1186/s13223-022-00755-2

**Published:** 2023-01-17

**Authors:** Max Moloney, Geneviève Digby, Madison MacKinnon, Alison Morra, David Barber, John Queenan, Samir Gupta, Teresa To, M. Diane Lougheed

**Affiliations:** 1 Asthma Research Unit, Kingston Health Sciences Centre, Kingston, ON Canada; 2 Division of Respirology, Department of Medicine, Queen’s University, Kingston, ON Canada; 3 Department of Family Medicine, Queen’s University, Kingston, ON Canada; 4 Canadian Primary Care Sentinel Surveillance Network (Eastern Ontario Network), Kingston, ON Canada; 5 Division of Respirology, Department of Medicine, St. Michael’s Hospital, Toronto, ON Canada; 6 Li Ka Shing Knowledge Institute, St. Michael’s Hospital, University of Toronto, Toronto, ON Canada; 7 Child Health Evaluative Science, Research Institute, The Hospital for Sick Children, Toronto, ON Canada; 8 Dalla Lana School of Public Health, University of Toronto, Toronto, ON Canada

## Abstract

**Background:**

Viable knowledge translation (KT) strategies are increasingly sought to improve asthma diagnosis, particularly in primary care. Despite this understanding, practical KT tools to support primary care practitioners are not widely available. Electronic medical records (EMRs) offer an opportunity to optimize the diagnosis and surveillance of chronic diseases such as asthma, and support quality improvement initiatives that increase adherence to guideline-recommended care. This review aims to describe the current state of electronic KT electronic tools (eTools) and surveillance systems for asthma and identify opportunities to increase adherence to asthma diagnostic guidelines by implementing digital KT eTools.

**Methods:**

Systematic literature searches were conducted on Ovid MEDLINE that included the search terms: asthma, asthma diagnosis, asthma surveillance, electronic health records, translational medical research, quality improvement, professional practice gaps, and primary health care published in the previous 10 years. In total, the searches returned 971 articles, 163 of which were considered relevant and read in full. An additional 28 articles were considered after reviewing the references from selected articles. 75 articles were included in this narrative review.

**Results:**

Established KT eTools for asthma such as electronic questionnaires, computerized clinical decision support systems (CDSS), chronic disease surveillance networks, and asthma registries have been effective in improving the quality of asthma diagnosis and care. As well, chronic disease surveillance systems, severe asthma registries, and workplace asthma surveillance systems have demonstrated success in monitoring asthma outcomes. However, lack of use and/or documentation of objective measures of lung function, challenges in identifying asthma cases in EMRs, and limitations of data sources have created barriers in the development of KT eTools. Existing digital KT eTools that overcome these data quality limitations could provide an opportunity to improve adherence to best-practice guidelines for asthma diagnosis and management.

**Conclusion:**

Future initiatives in the development of KT eTools for asthma care should focus on strategies that assist healthcare providers in accurately diagnosing and documenting cases of asthma. A digital asthma surveillance system could support adherence to best-practice guidelines of asthma diagnosis and surveillance by prompting use of objective methods of confirmation to confirm an asthma diagnosis within the EMR.

## Background

Globally, the number of people diagnosed with asthma is over 340 million and has continually increased over a 10-year period [1]. Asthma is diagnosed based on a combination of patient history, physical examination, and objective tests. Asthma poses a significant burden on individuals and the health care system at-large. As the prevalence of asthma increases, the burden of asthma on healthcare systems around the world will also increase given that individuals with asthma use significantly greater health care resources than those without asthma, have a poorer quality of life, and have a higher chance of suffering from mental illness [2–5].

A major contributor to the burden of asthma on individuals and the healthcare system is that gaps exist between the published guidelines for asthma diagnosis and actual strategies for diagnosis used in primary care [4, 6]. Although standards for asthma diagnosis are well established, less than half of individuals diagnosed with asthma have a confirmed diagnosis through the use of objective measurements of pulmonary function within two years of their original diagnosis [7]. Other challenges in the diagnosis of asthma include differentiating asthma from other respiratory conditions given the vast differential diagnosis for characteristic asthma symptoms [8]. These issues are compounded by a limited number of validated knowledge translation (KT) initiatives that could potentially support practitioners in the diagnosis and surveillance of asthma patients in primary care [9]. Incorporation of data element standards outlined in the Pan-Canadian Respiratory Standards Initiative for Electronic Health Records (PRESTINE) and specific indicators for asthma in primary care have created the possibility for improved asthma KT eTools in primary care by adopting these data standards [10, 11].

KT is a term that describes the process of implementing the results of research into practice [12]. The process of KT has been organized through the development of the Knowledge to Action (KTA) Framework put forward by Graham et al. [13]. KTA is broken down into two distinct phases: Knowledge Creation and Knowledge Action. The Knowledge Creation phase involves analysis of the available research on the topic of interest from primary studies to systematic reviews. The Knowledge Action phase occurs simultaneously or after Knowledge Creation. Knowledge Action involves synthesizing available research, identifying potential barriers, and implementing interventions [13].

KT has become a priority for many stakeholders with an interest in improving asthma diagnosis and care as its approach to integrating research from various fields provides an opportunity to create new tools to assist practitioners, particularly in primary care [14, 15]. KT initiatives are required for quality improvement in asthma care as research findings must be translated into usable interventions that create actionable behaviour change in physicians. By following the KTA framework, researchers can ensure novel research findings are implemented effectively to reach more health care providers and improve decision making [13].

Despite this understanding, practical KT tools to support primary care practitioners are not widely available. Electronic medical records (EMRs) offer an opportunity to improve practitioner performance and support quality improvement efforts by accurately identifying patients with asthma. This review aims to describe the current state of KT initiatives for asthma, to assess the limitations of KT tools for asthma and identify opportunities for how to improve asthma diagnosis and surveillance through digital innovations.

### Methods

Systematic literature searches were conducted on Ovid MEDLINE and Ovid MEDLINE Daily Epub Ahead of Print, In-Process & Other Non-Indexed Citations. The first search criteria included the key words: “asthma”, “validation study”, and “electronic health records”. Supplemental searches included the terms: “translational medical research”, “asthma surveillance system” “asthma definition”, “primary health care”, “quality improvement”, and “professional practice gaps”. The search criteria included original articles; had a date restriction limiting results from 2012 to 2022; and was limited to articles in English. Studies referenced in research articles from the original literature search were also identified. Articles were reviewed if they fit within one of four themes: (1) electronic KT tools for asthma; (2) electronic asthma surveillance; (3) quality improvement of asthma diagnosis in primary care; and (4) gaps in asthma diagnosis and surveillance in primary care.

In total, the search returned 971 articles, of which each title and abstract were reviewed. Following review of title and abstract, a total of 163 articles were considered sufficiently relevant to review and were subsequently read in full. Of the 163 articles read in full, 43 were met one of the themes identified. An additional 28 articles were included from references of the articles read in full. After exclusion of articles that had outdated information and were either not relevant to the topic or contained redundant information, 75 articles were included.

## Results

### Electronic knowledge translation tools for asthma diagnosis

There are two primary categories of KT eTools for the diagnosis of asthma: electronic questionnaires and clinical decision support systems (CDSS).

#### Electronic questionnaires

Electronic questionnaires can be used in a variety of health care settings to gather information important to asthma diagnosis and surveillance [16, 17]. In clinical practice, questionnaires are generally used as a standardized assessment tool prior to meeting with a clinician. Due to their ease of use and low cost, questionnaires have been used to estimate the prevalence of asthma in children and adults around the world [18]. These questionnaires collect information including symptoms, asthma control, and quality of life [19, 20]. A limitation of electronic questionnaires is the reliance on patients to accurately self-report symptoms and exacerbations, which introduces recall and other biases that could impact validity [21, 22]. Another challenge of implementation is the limited uptake of questionnaires by clinicians and patients who are provided electronic questionnaires [23, 24]. Overall, questionnaires have been shown to be effective for improving the diagnosis of asthma by gaining additional insight into patient symptoms and history, however difficulties related to accuracy and uptake of these electronic questionnaires remain.

#### Clinical decision support systems

There are currently several ongoing initiatives utilizing clinical decision support systems (CDSS) to improve asthma diagnosis. A CDSS is a KT eTool designed to improve health care delivery by enhancing medical decisions with targeted clinical knowledge, patient information, and other health data [25]. A currently operational CDSS for asthma is the Electronic Asthma Management System (eAMS) in use in Toronto, Ontario. eAMS is a computerized CDSS aimed at addressing major care gaps for adult asthma and has demonstrated effectiveness in improving rates of assessment of asthma control levels and other metrics important to diagnosis of asthma in Canada [26]. The eAMS collects data through a pre-visit questionnaire that patients complete on a tablet in the office and includes questions on symptom control, medication usage, triggers, and allergies. This information is then inputted into a CDSS system unique to eAMS which then creates an output for the physician highlighting asthma control status, medication changes recommendations, and an asthma action plan. These outputs are integrated into the clinician facing EMR system for use during the patient consultation. eAMS is a KT eTool that has demonstrated effectiveness in improving asthma action plan delivery, assessments of asthma control, and prescription of asthma medications [26]. The findings of the eAMS study demonstrate the potential for KT eTools to support quality improvement of asthma diagnosis and management in primary care.

Other CDSSs have been created in various jurisdictions over the past decade and have demonstrated the potential for eTools to improve asthma diagnosis and outcomes [27]. For example, AsthmaCritic developed in the Netherlands is a guideline-based provider critiquing system that uses information from EMRs to monitor and change practitioner behavior [28]. AsthmaCritic has demonstrated the ability to improve the number of PFTs administered and improve adherence to best-practice guidelines a randomized controlled trial [28]. Another KT eTool to assist in diagnosis is the Severe Asthma Algorithm (SAA), which assists health care providers in diagnosing severe asthma by using standardized data elements and decision support that prompts adherence with best practice guidelines for severe asthma diagnosis within an EMR [29]. Other CDSS studies have incorporated the development of algorithms using machine learning principles, which have the potential to uncover new risk factors and triggers of asthma using EMR data in an effort to improve diagnosis [30]. Despite their potential, CDSS for asthma experience similar limitations to questionnaires, including limited practitioner uptake and lack of utilization by patients [31, 32].

#### Potential of electronic knowledge translation tools from a quality improvement perspective

KT eTools for asthma diagnosis have demonstrated effectiveness as a tool for quality improvement. Considering these interventions within the Hierarchy of Intervention Effectiveness, a framework that rates interventions related to human behaviour lower on a scale of effectiveness compared with system-focused interventions, both electronic questionnaires and CDSS are best categorized as people-focused interventions [33]. People-focused interventions require individuals to make conscious decisions to both use the intervention and subsequently alter their behaviour based on the information provided by the intervention to impact quality of care. As such, KT eTools that are people-focused are likely best used as components within larger system-focused eTools to improve asthma diagnosis. An opportunity exists to build upon these interventions by using EMR data to support system-focused interventions for quality improvement of asthma diagnosis in primary care.

### Electronic knowledge translation tools for asthma surveillance

There are four primary categories of KT eTools for the surveillance of asthma: chronic disease surveillance networks, asthma registries, asthma quality of care monitoring systems, and work-related asthma surveillance systems.

#### Chronic disease surveillance networks

Chronic disease surveillance networks are a category of asthma KT eTool that exist at international, national, and regional levels [34]. An example of a chronic disease network is the Canadian Primary Care Sentinel Surveillance Network (CPCSSN) [35]. CPCSSN gathers information from physician billing codes, emergency department visits, and administrative data to facilitate standardized estimates of the incidence, prevalence, and outcomes related to various chronic diseases and are used by governments and researchers. Although CPCSSN collects data on a variety of chronic diseases, it does not currently collect data on adult asthma [35]. An example of a chronic disease surveillance network for asthma is the Ontario Asthma Surveillance Information System (OASIS) [36]. OASIS uses a previously validated asthma case definition derived from hospital administrative data in Ontario and provides a population-based longitudinal surveillance system for asthma. OASIS data is used to provide estimates of asthma incidence and prevalence, measures of asthma-related morbidity, mortality, health services use, and provider practice patterns using hospital administrative data to track quality of care over time [36]. Chronic disease surveillance networks are often limited by their source of data and restricted criteria for defining a case of the condition [37]. While limited criteria from health administrative data such as billing codes are sufficient for chronic diseases such as diabetes, for more complex and heterogenous conditions such as asthma, health administrative data often do not accurately reflect when and how diagnoses were made [38]. These chronic disease surveillance networks that are based on health administrative data have limitations on the amount and quality of data they can leverage to improve asthma diagnosis and surveillance.

#### Asthma registries

Registries have been effective tools to collect uniform data to evaluate specific outcomes for a population defined by a particular disease. Registries differ from surveillance networks in that registries use data that is voluntarily provided and entered while surveillance networks make use of pre-existing data. The scope of asthma registries has been limited to severe asthma, which represents a minority of total asthma cases in Canada and worldwide [39, 40]. Severe asthma registries gather anonymous, longitudinal, real-life data for patients with severe asthma. To date, there are over 25 severe asthma registries, the majority of which operate at a national level and contribute information to the International Severe Asthma Registry (ISAR), the Severe Heterogenous Asthma Research collaboration, Patient-centered (SHARP), or the Severe Asthma Research Program (SARP) [41–43].The availability of health information varies greatly between databases [41]. In addition to ISAR, SHARP, and SARP there are several local and regional initiatives aimed at creating registries for cases other than severe asthma however, to date there remains no asthma registry for general asthma patients, only severe asthma patients [44]. The result of this is a lack of centralized information worldwide for general asthma patients, which creates a significant challenge to monitor the state of asthma at a national or international level.

#### Asthma quality of care monitoring systems

Quality of care monitoring systems for asthma are emerging as useful eTools for KT. Audit and feedback systems for quality improvement of chronic disease can be effective in creating behaviour changes in health care providers when successfully implemented and can monitor quality of care over time [45]. There are currently several initiatives centered around creating quality of care monitoring systems for asthma in different forms. The Asthma Care Map (ACM) developed by the Lung Health Foundation (formerly the Ontario Lung Association) and used in the Primary Care Asthma Program (PCAP) is a paper KT tool for asthma quality of care [46] that is currently being adapted to integrate within primary care EMRs. Advancements in computing and data quality within EMRs have given rise to the potential for electronic systems to aid in the surveillance of chronic diseases, with the end goal of improving patient outcomes [47]. One example of a management and monitoring system that can be used for quality improvement is the Asthma Management and Outcomes Monitoring System (AMOMS) [48]. AMOMS is a point-of-care charting tool the prompts providers to document care in accordance with best practice guidelines. In doing so, AMOMS collects data from the patient and physician that can be extracted and used to support performance measurement, benchmarking, and quality improvement. Another quality of care monitoring system for asthma developed by Tomasallo et al. (49) found their EMR-based asthma surveillance system can be used to estimate the prevalence of asthma in both adults and children across time and was able to identify patients at risk of asthma in 50% more cases than traditional telephone surveys [49]. Despite the potential for quality of care monitoring systems to improve adherence to best-practice guidelines and patient outcomes, this literature review did not identify any quality of care monitoring systems for asthma that have achieved scale at a national or international level.

#### Work-related asthma surveillance systems

There are several surveillance systems dedicated to work-related asthma (WRA). WRA surveillance programs have been active for over 20 years in jurisdictions ranging from local to national in scale [50, 51]. Similar to chronic disease surveillance networks, there are also occupational disease surveillance systems that record the incidence, prevalence, and outcomes of WRA [52]. WRA surveillance systems have been effective in supporting individuals with WRA; however, their scale is often limited by the source of the data and represents only a fraction of total asthma cases. In addition, previous efforts to develop workplace asthma surveillance systems have relied on practitioner reporting and have reported low uptake rates.[53] Due to their small scale and limited sources of data, WRA surveillance systems are unlikely to scale to the national or international level.

#### Potential of electronic knowledge translation tools for asthma surveillance from a quality improvement perspective

Within the Hierarchy of Intervention Effectiveness, system-focused interventions have been demonstrated to be more effective in producing behaviour change in health care providers.[33] Several of the described KT eTools for asthma surveillance bring a system-level component through standardized data collection, computerized registries, and automated data reporting. However, these eTools are primarily used for research purposes and population health analyses, and are not necessarily used at the point of care to drive change on an individual patient level. An opportunity exists to utilize improved EMR data and advances in computing to support surveillance interventions for quality improvement of asthma care at the patient level.

## Discussion

### Opportunities for KT eTools in diagnosis and surveillance of asthma

To date, the majority of KT eTools for asthma have required users to make conscious changes to their behaviours in order to use the tools, requiring change in daily routines and practices. This serves as a significant barrier to the adoption of eTools for asthma diagnosis and surveillance. Future KT eTools should leverage improvements in EMRs to reduce cognitive load on physicians, automate decision making, and be embedded within the EMR to facilitate adherence to best-practice guidelines for asthma diagnosis and surveillance using a system-focused approach. The following is a summary of opportunities in the development of KT eTools to improve asthma diagnosis and surveillance.

#### EMRs

An excellent opportunity exists for asthma KT eTool development by leveraging EMRs to support evidence-based diagnosis, surveillance, and quality improvement [48, 54]. EMR-based tools also have the added benefit of potentially reducing friction between practitioners and the eTool through automation, a system-level intervention as per the Hierarchy of Intervention Effectiveness. This opportunity to improve asthma diagnosis and surveillance is most relevant to primary care practitioners, who face numerous challenges in keeping up with updated guidelines and effectively integrating them into their practice. As a result, KT eTools that involve decision support may improve adherence to evidence-based guidelines and improve outcomes [55].

To improve the accuracy of asthma diagnoses and the overall quality of asthma patient care, KT eTools within EMRs should be developed that reinforce evidence-based guidelines for asthma diagnosis, particularly in primary care. The symptoms of asthma are similar to several other obstructive lung diseases, in particular chronic obstructive pulmonary disease (COPD) and asthma-COPD overlap syndrome (ACOS). This adds another layer of difficulty in developing an EMR case definition that detects cases of asthma and is able to discriminate asthma from other respiratory conditions [56]. To account for these challenges, new KT eTools should support asthma diagnosis to include objective evidence of asthma confirmation through PFTs within the EMR [57]. Additionally, information to support quality improvement should be optimized to reduce the cognitive load on the health care provider, which has proven to increase the effectiveness of surveillance systems and EMR tools in practice [58]. Fully embedding a new KT eTool within the EMR is an excellent opportunity to facilitate adherence to best-practice guidelines for asthma diagnosis and surveillance.

#### Surveillance tools

KT eTools for asthma surveillance present a unique opportunity to improve provider diagnosis of asthma by promoting adherence to best-practice guidelines. Emphasis should be placed on addressing specific gaps in asthma diagnosis, such as the lack of objective measurements to confirm asthma. Surveillance eTools have the ability to provide a multitude of surveillance metrics that practitioners can track to improve their practices [59]. Surveillance tools also have the ability to prompt actionable, individualized feedback to facilitate adherence to best practice guidelines [60]. An optimal surveillance system to improve the quality of asthma care necessitates an accurate diagnosis of asthma to monitor patients over time and effectively change provider behaviour. With an accurate diagnosis of confirmed asthma, surveillance tools have the potential to greatly improve adherence to best-practice guidelines.

#### PRESTINE data elements

There are a variety of KT eTools that have been developed for diagnosis, education, and management of asthma that have been shown to improve outcomes for individuals with asthma despite data source limitations. These previous innovations or new KT eTools have the potential to be improved by utilizing data element standards outlined in the Pan-Canadian Respiratory Standards Initiative for Electronic Health Records (PRESTINE) [10]. PRESTINE is a set of data elements and definitions recommended by experts for inclusion in EMRs to support primary, secondary, and tertiary care for respiratory conditions, including asthma to enable monitoring, benchmarking, and performance evaluation. Adopting the PRESTINE data elements for asthma into primary care have created the possibility for new asthma eTools in primary care [11]. Adoption of these data standards into new KT eTools and EMRs could be beneficial in allowing eTools to distinguish between confirmed and suspected asthma.

### Limitations of KT eTools for asthma diagnosis and surveillance

**Fig. 1:**
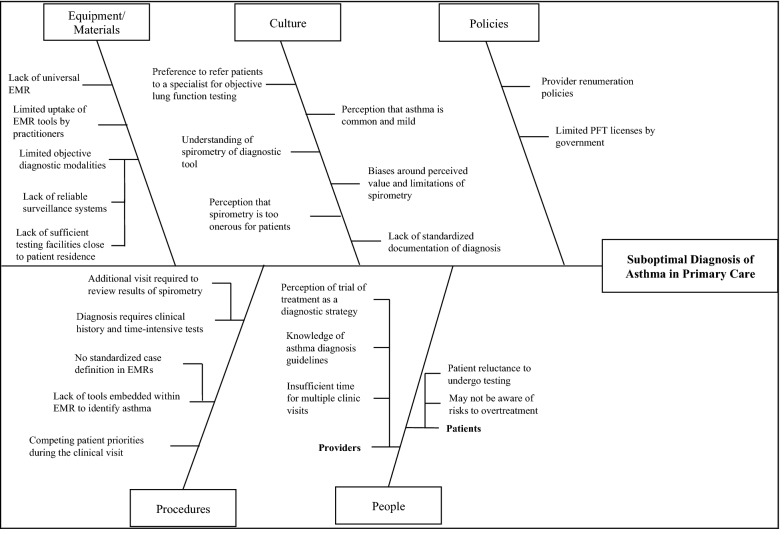
Barriers to optimal asthma diagnosis in primary care

In order to consider the specific limitations of KT eTools in the diagnosis and surveillance of asthma, we need to understand the broader system factors that lead to suboptimal diagnosis of asthma in primary care. Previous publications, including a recent publication by Yamada et al. have identified several such barriers that can be categorized in the following themes: knowledge, skills, social/professional role and identity, beliefs about capabilities, reinforcement, intentions, goals, memory and decision processes, environmental context and resources, social influences, emotions, and behavioural regulation [61]. Figure 1 provides a root cause analysis for suboptimal asthma diagnosis in primary care, outlining several of these key factors. These barriers stem from a variety of sources, including availability of equipment and materials, culture, government policies, process and procedures for diagnosing patients, and people factors, all of which contribute to barriers to diagnosis of asthma in primary care (Fig. 1). We will consider these barriers in more detail below, in order to identify the potential for KT eTools to contribute to the necessary mitigating strategies to improve quality of asthma diagnosis and care.

#### Lack of confirmation of asthma diagnosis

KT eTools for surveillance rely first on ensuring patients have an accurate diagnosis of their condition by a health care provider. The gold-standard definition of asthma, as outlined by the Canadian Thoracic Society and Global Initiative for Asthma, requires objective measurement of lung function or airway responsiveness using pulmonary function tests [57, 62, 63]. Reliance on clinical history without the support of objective measurements leads to misdiagnosis of asthma in 33% of cases [64]. While rates of use of pulmonary function tests vary widely based on jurisdiction, objective measurements are not widely utilized by primary care providers in asthma diagnosis [7]. Underutilization of objective measurements to diagnose asthma in primary care sites can lead to both underdiagnosis and overdiagnosis of asthma [65]. Individuals who suffer from asthma but have not received a diagnosis continue to struggle with symptom management and further contribute to the burden of asthma on the health care system through underdiagnosis [66]. Likewise, overdiagnosis of asthma also compounds the effects on the health care system through unnecessary patient visits and medication prescriptions [65].

In primary care, one of the greatest contributors to misdiagnosis stems from this lack of objective pulmonary function measurement in the process of diagnosing a patient with asthma [57, 62]. The use of objective measurements such as spirometry, methacholine challenge tests, and exercise challenge tests are crucial to ensuring the confirmation of an asthma diagnosis. Without completion of objective measures confirming asthma diagnoses, the accuracy of EMR data for patients labelled with asthma may not be valid. This creates barriers for researchers in creating KT eTools for asthma surveillance as many charts billed for asthma are in fact suspected asthma and not confirmed asthma.

#### Gaps in case definitions of asthma in EMRs

The gold-standard definition for the diagnosis of asthma is well established and provides clear guidance for practitioners to make an accurate diagnosis of asthma for patients with a clinical suspicion of the condition. There have been several attempts to translate the evidence-based clinical definition of asthma into a case definition to incidences of asthma in EMRs, however, no consensus has been reached [67]. Al Sallakh et al. (67) conducted an extensive review of attempts to define asthma using electronic health record data [67]. The review analyzed a total of 76 case definitions to identify asthma in EMRs and found significant heterogeneity in the case definitions proposed. This review found that for case definitions to be effective, they must be tailored to the EMR environment in which they function and consider the charting techniques of the practitioners who use the EMR. In Canada, there have been recent attempts to create a case definition for asthma suitable for Canadian EMR vendors and primary care practitioners. Previous efforts to create and validate case definitions for asthma have come from a single EMR or restricted data environments [68, 69]. Xi et al. (68) proposed a variety of case definitions using similar search fields with the addition of a search for asthma in the free text portion of the EMR and found a case definition of asthma that had a sensitivity of 90.2%, and a specificity of 83.9% [68]. Another recent publication from Cave et al. (69) conducted a study to validate a case definition for asthma using data from the Southern Alberta Primary Care Research Network, a node of the CPCSSN (SAPCReN-CPCSSN) [69]. The authors created a case-finding algorithm using a combination of search fields from the EMR including billing information, recorded encounter diagnosis information, and information inputted into a health condition field within the EMR. Cave et al. (2020) compared their algorithm against expert physician review of patient charts and found a sensitivity of 83.3% and specificity of 99.3%. Case definitions for asthma in EMRs have been proposed however, the limits of their generalizability remain unknown, as attempts have been limited to single EMRs in single jurisdictions and restricted data environments that do not have the ability to use all available data to make an accurate diagnosis.

#### Gaps in validation of case definitions for asthma

The reporting and validation of proposed case definitions within EMRs are critical to be able to draw reliable conclusions from the results of studies that derive their data from EMRs [70]. Determining the validity of the case definition of diseases such as asthma is more challenging than many other chronic conditions [71]. Asthma case definitions require a complex combination of symptom assessment, pulmonary function tests, and practitioner interpretation of the objective measurements. Meanwhile, other chronic conditions such as diabetes can be confirmed through a single blood test. Previous research has suggested several methods for creating and validating case definitions, including manual validation, comparison to external databases, comparison of rates in similar populations, and machine learning algorithms [72]. The preferred method for ensuring the accuracy of reference diagnoses when establishing cases of asthma is manual chart review, however manual reviews are labour-intensive and often involve additional methods to protect confidentiality that constitute barriers to this methodology [46]. Several studies assessed in this review contained minimal or no information on the methodology used in validating their proposed case definition for asthma. Overall, the information available on the methodology and results of previously published case definition validation studies for asthma is suboptimal. Thus, additional work is required to appropriately validate a case definition for asthma in which the methodology can be replicated.

#### Limitations of data sources

An important aspect of improving asthma outcomes is high quality datasets from which to derive health information to inform KT eTools for asthma diagnosis and surveillance [73]. A critical barrier to the scalability of asthma eTools are sources of accurate data. Most KT eTools described in this review originate from the local context in which they were created. This can limit the scalability of the eTools to other settings. The majority of KT eTools that have been developed are only functional in one EMR environment or derive information from health administrative data, which is limited in detail [38, 67–69]. While these tools may be effective in a local context, the inability to scale tools across multiple EMRs and the limited information provided by health administrative data create limitations on the ability of these KT eTools to scale to the national and international level. Furthermore, the quality of data available for eTools within the data sources serves as another potential limitation in the development of eTools for asthma. Inconsistencies in practitioner charting behaviours for asthma, particularly in primary care, can have a negative impact on the quality of data that informs eTools [74]. Further studies have demonstrated the high variability and generally low quality of information inputted into various EMR fields, affecting the completeness of data available to these eTools [75]. Adopting PRESTINE data elements in EMRs could improve upon the data sources currently available KT eTools.

#### Setting of KT eTool implementation

Another barrier to the development of KT eTools for asthma is that proposed KT eTools are often designed for different purposes depending on the health care setting or the purposes of the original study from which the definition was derived [67]. Many studies designing case definitions for asthma to incorporate into eTools have a wide range of sensitivity, specificity, positive predictive value, and negative predictive value. This variation and how it affects the selection of a case definition is important because a case definition with a high sensitivity is key to identifying all cases of asthma within a database, but if excluding non-cases is the area of interest then a high specificity is more important. Likewise, there is a trade-off between PPV and NPV in which one statistic can be more important than the other depending on whether the aim of the study is to determine true positives or true negatives. As a result, testing multiple case definitions to determine the case definition for asthma that is best suited for KT intervention which it will be used for is crucial to ensure the best case definition for the purpose of the intervention is selected.

#### KT eTools from a quality improvement perspective

A quality improvement approach to optimizing asthma diagnosis in primary care requires tackling the numerous root causes identified above. If implemented at a system-level, in a standardized, automated, and computerized manner, the KT eTools outlined in this paper have the potential to target several of these root causes, particularly where decision making at the physician level is required. Sophisticated KT eTools could include automation and forced functioning to ensure spirometric confirmation of an asthma diagnosis. However, KT eTools alone will not be able to overcome all of the barriers to confirmatory testing with spirometry, such as availability of testing facilities or policy level factors that impact decision making. Yet, available data from existing KT eTools, registries, surveillance systems, and quality of care monitoring systems can be used to leverage policy level changes that could further alleviate barriers to optimal asthma diagnosis in primary care. Ultimately, future KT eTools and the strategies by which they are implemented, must be leveraged to address the identified barriers to improve patient outcomes in asthma.

## Conclusion

This review identifies opportunities to improve the accuracy of asthma diagnosis and surveillance through the use of KT eTools to improve the quality of asthma care. The key barriers to effective KT for asthma using EMR data are lack of documentation of confirmation of an asthma diagnosis, challenges related to creating a valid EMR case definition for asthma in the absence of this documentation, and the limitations of data sources that can inform KT eTools. Limited access to and use of pulmonary function tests and specialist consultation contribute to misdiagnosis and suboptimal management. Existing KT tools for asthma have been limited in scope and many fail to address barriers and challenges in primary care, where the majority of asthma diagnoses are made. As a result, future research should focus on KT initiatives that integrate surveillance systems that can be used with multiple EMR vendors with system-level quality improvement strategies to improve health care provider adherence with guideline-recommended care on a national and international scale. By promoting and documenting accurate asthma diagnoses, KT tools and surveillance systems based on reliable EMR case definitions can be used for performance evaluation and optimization of asthma care.

## Data Availability

The datasets used and/or analysed during the current study are available from the corresponding author on reasonable request.

## Abbreviations

ACM: Asthma Care Map
AMOMS: Asthma Management and Outcomes Monitoring System
ARU: Asthma Research Unit
CDSS: Clinical Decision Support System
CPCSSN: Canadian Primary Care Sentinel Surveillance Network
CTS: Canadian Thoracic Society
eAMS: Electronic Asthma Management System
e-API: Electronic Asthma Performance Indicator Reporting System
eAQLQ: Electronic Asthma Quality of Life Questionnaires
EMR: Electronic medical record
eTool: Electronic tool
KT: Knowledge translation
KTA: Knowledge to action
OASIS: Ontario Asthma Surveillance Information System
PAAF: Provider Asthma Assessment Form
PC-API: Primary Care Asthma Performance Indicators
PFT: Pulmonary function test
PRESTINE: Pan-Canadian Respiratory Standards Initiative for Electronic Health Records
WRASQ(L): Work-Related Asthma Screening Questionnaire-Long Version
